# Understanding vaccination against human papillomavirus (HPV): knowledge and perceptions among parents or guardians and healthcare workers, Germany, 2023

**DOI:** 10.2807/1560-7917.ES.2026.31.41.2500910

**Published:** 2026-10-15

**Authors:** Nora Katharina Schmid-Küpke, Elisa Wulkotte, Yvonne Bichel, Heike Kramer, Johannes Lachmann, Andrea Mais, Philipp Schmid, Jule Marie Schmitz, Ole Wichmann, Anja Takla

**Affiliations:** 1 Immunization Unit, Robert Koch Institute, Berlin, Germany; 2 Charité - Universitätsmedizin Berlin, corporate member of Freie Universität Berlin and Humboldt-Universität zu Berlin, Berlin, Germany; 3 Medical Society for Health Promotion (registered association), Hamburg, Germany; 4 Centre for Language Studies, Radboud University, Nijmegen, The Netherlands; 5 Institute for Planetary Health Behaviour, University of Erfurt, Erfurt, Germany

**Keywords:** paediatricians, counselling, communication, survey, training programs, recommendation, medical assistants

## Abstract

**BACKGROUND:**

Vaccination against human papillomavirus (HPV) is effective against certain types of cancer. Despite longstanding vaccination recommendation, HPV vaccination has the lowest coverage among all recommended vaccines for children and adolescents in Germany, with notable differences between sexes.

**AIM:**

We aimed to investigate attitudes towards vaccination against HPV and HPV-related knowledge among parents or guardians and healthcare workers (HCW) in Germany, alongside vaccination intentions and determinants in parents/guardians and vaccination counselling in HCWs.

**METHODS:**

In autumn 2023, we conducted telephone interviews with 1,508 parents/guardians of children and adolescents aged 9–14 years and 503 HCWs from paediatric practices. We used weighting and analysed the responses with t test and ANOVA.

**RESULTS:**

Overall, 34.5% children of the interviewed parents/guardians were already vaccinated against HPV, while 24.8% of parents/guardians reported a high intention, 5.6% opposed to vaccination and 35.2% were considered vaccine hesitant. Parents/guardians and HCWs considered vaccination of females more important than of males (p < 0.001). Parents/guardians exhibited knowledge gaps, for example, by overestimating alternative measures (e.g. condom use) for cancer prevention; HCWs expressed uncertainties about the risk of HPV infection. Vaccination decisions were most strongly influenced by the paediatricians the parents/guardians consulted. Although 97% of HCWs reported recommending the vaccine, only 43.7% of parents/guardians recalled receiving a recommendation.

**CONCLUSION:**

These findings highlight the need for tailored vaccination communication, alongside timely, active physician recommendations and appropriate education to support HPV vaccination decisions.

**Key public health message**

**What did you want to address in this study and why?**

Human papillomavirus (HPV) infection is very common and can cause cervical cancer and some other types of cancer. Vaccination against HPV is the most effective way to prevent HPV infections, yet many teenagers in Germany remain unvaccinated. We aimed to investigate attitudes to and knowledge about HPV and HPV vaccination among parents or guardians of 9–14-year-olds and healthcare workers in Germany.

**What have we learnt from this study?**

More than one in three parents or guardians in Germany were hesitant about HPV vaccination for their child. Parents/guardians mainly relied on doctors when deciding about HPV vaccination, yet fewer than half remembered receiving a vaccination recommendation from a healthcare worker. Parents/guardians and healthcare workers perceived HPV vaccination as more important for girls than for boys.

**What are the implications of your findings for public health?**

Increasing uptake of HPV vaccination among children and adolescents is important to prevent HPV-related cancer. Extra effort is needed to reach vaccine-hesitant parents. Healthcare workers, as trusted communicators, play a central role in vaccination decisions and should be further supported in communicating effectively and actively recommending the HPV vaccination at the recommended vaccination age, regardless of sex.

## Introduction

Human papillomavirus (HPV) infections are highly prevalent, with most sexually active people becoming infected at least once over their lifetime due to the circulation of multiple HPV types without cross-protection and limited natural immunity after infection [1]. While typically transient, persistent HPV infections can progress to dysplasia, precancerous lesions and carcinomas. In Germany, a country with 84 million inhabitants, ca 10,000 people develop HPV-related cancers each year, including approximately 7,000 women [2]. Globally, oropharyngeal carcinomas have risen in recent decades, particularly among men [3]. Most HPV-related diseases could be prevented by vaccination in childhood and early adolescence.

Since 2007, the Standing Committee on Vaccination (STIKO) in Germany has recommended HPV vaccination for girls and since 2018, for all children and adolescents aged 9–14 years [4]. Initial uptake among girls increased steadily but has stagnated in recent years: since 2021, vaccination coverage among 15-year-old girls for a complete HPV vaccination series has hovered around 54% [5]. Uptake among boys has also slowed: 5 years after the recommendation was endorsed, HPV coverage was 34% in 2023, still 20 percentage points lower than among girls of the same age. Moreover, by 2023, there were considerable regional differences: in some districts, only 27% of girls and 9% of boys were fully vaccinated, while in others, coverage reached 81% and 72%, respectively [5]. Overall, HPV vaccination coverage remains substantially lower than that of the other routine childhood vaccinations [5]. One contributing factor to the low and stagnating uptake in Germany could be vaccine hesitancy, defined as the delay in acceptance or refusal of vaccination despite availability of vaccination services [6]. Drawing on health behaviour theories, hesitancy can be driven by negative attitudes towards vaccination, low perceived social norms to get vaccinated, limited self-efficacy in getting vaccinated, knowledge gaps and a lack of trust in actors [7].

Given its considerable preventive potential, the World Health Organization (WHO) and the European Commission have set the target of achieving 90% HPV vaccination coverage among girls by 2030 and substantially increasing uptake among boys [8,9], as part of broader efforts to eliminate cervical cancer as a public health problem. To achieve this goal, vaccination coverage in Germany as in many other European countries [10] must increase considerably.

The ‘Intervention Study to Increase HPV Vaccination Coverage in Germany’ (InveSt HPV) supports this effort [11]. The project examines how healthcare workers (HCW) could be supported through targeted communication training, particularly in conversations with parents uncertain about HPV vaccination. In Germany, most childhood and adolescent vaccines (75–93%) are administered in private practices with paediatricians [12]. Although physicians are legally responsible for providing vaccination counselling, other HCWs also play an important role in vaccination-related communication in paediatric practices. They often serve as the first point of contact for parents, may provide information about vaccinations, check vaccination booklets and, when delegated by a physician, administer vaccines. Previous studies have identified lack of information and the absence of an HCW recommendation as important barriers to HPV vaccination uptake [13-15]. Considering the perspectives of HCWs as vaccination providers and of parents and guardians as decisionmakers allows for a better understanding of their interaction. Against this background, we aimed to explore attitudes towards HPV vaccination and HPV vaccination recommendations among parents/guardians of children and adolescents and HCWs in paediatric practices.

## Methods

### Participants and recruitment

In autumn 2023, we conducted telephone surveys with parents/guardians of children and adolescents and HCWs in Germany using a fully structured, computer-assisted telephone interview (CATI) questionnaire. Hereafter, the term parents refers to both parents and legal guardians.

Parents with at least one child aged 9–14 years were eligible for inclusion. Overall, the target sample size was 1,500 parents. The target sample size was determined pragmatically to provide sufficiently precise descriptive estimates overall and for parents of a 9–14-year-old. Following best practices [16], a sample size of ca. 384 respondents is sufficient to estimate population proportions with a 95% confidence level for large populations such as the German population of parents.

This sample was drawn using the German Arbeitskreis Deutscher Markt- und Sozialforschungsinstitute (ADM) telephone sampling system which enables representative sampling of private households and provides statistical background information [17]. As there is no complete registry of privately used phone numbers in Germany, the working group ADM telephone sampling provides a generated sampling frame that can be used by survey institutes to draw representative random samples of households reachable by landline and mobile phones. For this survey, we used a dual-frame approach, combining landline numbers (60%) and mobile numbers (40%). In total, 440,700 phone numbers were contacted. To identify parents of children or adolescents of the relevant age, eligibility was assessed during the initial contact phase. If two eligible target persons were present in the household, the respondent was selected using the Kish selection grid [18]. If there was more than one child in the relevant age group, one child was selected at random, and all questions referred to that child only. During the interview, additional screening questions were asked about the numbers and ages of children and adolescents in the household; depending on the responses, these questions could result in termination of the interview.

The target sample sizes in Bavaria and Bremen were 300 each, as these two federal states served as intervention regions for the aforementioned training due to their low HPV vaccination coverage.

For the survey of HCWs in paediatric practices, we aimed to reach a sample size of 500. Phone numbers were drawn from an address database of physicians and medical practices, restricted to paediatrics, and supplemented by internet searches using publicly available physician and practice directories. The two address lists were merged, checked for duplicate entries based on phone numbers and addresses, and cleaned. The resulting address file was quality-checked against available statistics on office-based paediatric practices in Germany. As the number of addresses was largely consistent with the available reference statistic, the address file was considered to provide broad coverage of the target population. A sample using simple random sampling method was then drawn from this cleaned address file. Practices were contacted until the predefined target of 500 respondents was reached, equally divided between physicians and other HCWs. In total, 5,451 phone numbers were contacted. Oversampling of intervention regions was not attempted, as we considered HCWs are difficult to reach for research purposes and the sample sizes needed for stratified analyses could not be achieved, particularly in Bremen, Germany's smallest federal state.

### Questionnaires

In the parent questionnaire, we asked about sociodemographic characteristics of the parent and their 9–14-year-old, knowledge of HPV and HPV vaccination, vaccination status and intentions for the child/adolescent, and determinants of vaccination behaviour (e.g. attitudes towards vaccination, normative beliefs, motivation to comply, perceived effectiveness, self-efficacy, trust) (Table 1). The HCW questionnaire included questions on vaccination consultations (e.g. frequency and recommendation behaviour), parental reactions to the HPV vaccination recommendation and HPV-related knowledge. The selection and interpretation of variables were guided by health behaviour theories, such as the theory of planned behaviour (norms, self-efficacy, attitude, intention) [19] as well as by insights from previous HPV studies on vaccine acceptance [20-24]. These theoretical and empirical sources were used as guidance for selecting determinants relevant to understanding vaccination decisions, parent-HCW interactions and the content of subsequent training interventions, rather than to formally test the theory of planned behaviour. The complete questionnaires are provided in Supplementary Tables S1 and S2.

**Table 1:** Overview of the variables included in a questionnaire about human papillomavirus, Germany, 2023

Measure and item	Response options
A. Parents or guardians with at least one child aged 9–14 years	
HPV vaccination intention	
If unvaccinated: Do you intend to have your child vaccinated against HPV?	1 = definitely not vaccinate; 2 = rather not vaccinate; 3 = undecided; 4 = rather vaccinate; 5 = definitely vaccinate
If vaccinated: Imagine you had to make the decision about HPV vaccination for your child again: Would you have your child vaccinated against HPV?	
Attitude towards HPV vaccination	
Vaccinating my child against HPV is…	1 = very unimportant; 2 = unimportant; 3 = neither unimportant nor important; 4 = important; 5 = very important 1 = very bad; 2 = bad; 3 = neither bad nor good; 4 = good; 5 = very good
Subjective norms regarding HPV vaccination^a^	
Normative beliefs about HPV vaccination of your child (for daughter/son: their own HPV vaccination): What do you expect – what is the opinion of your partner, your daughter/your son, parents you are friends with, your relatives and your child’s paediatrician?	−2 = definitely not vaccinate; −1 = rather not vaccinate; 0 = undecided; 1 = rather vaccinate; 2 = definitely vaccinate; 3 = not applicable/do not know
Motivation to comply: How motivated are you to follow the opinion of… (see above)?	1 = not at all motivated; 2 = rather not motivated; 3 = neither unmotivated nor motivated; 4 = rather motivated; 5 = fully motivated
Self-efficacy expectations regarding HPV vaccination	
To what extent would you be able to succeed in the following situations: • Having a good conversation about HPV vaccination with my child • Having a good conversation about HPV vaccination with my partner • Having a good conversation about HPV vaccination with general practitioner • Finding reliable information about HPV vaccination • Understanding information about HPV?	1 = not at all able to succeed; 2 = rather not able to succeed; 3 = partly able to succeed; 4 = rather able to succeed; 5 = fully able to succeed
Knowledge about HPV and HPV vaccination^b, c^	
In your opinion, does the statement apply, does it not apply, or do you not know?	Apply; Do not apply; Do not know
Perceived effectiveness of HPV vaccination^d^	
How do you rate the effectiveness of the following options for preventing cervical cancer: using condoms, having sexual intercourse with only one partner in life, healthy lifestyle (e.g. not smoking), HPV vaccination For women: Participation in cervical cancer screening?	1 = not at all effective; 2 = rather not effective; 3 = partly effective; 4 = rather effective; 5 = fully effective
Trust in actors involved in HPV vaccination	
Regarding HPV vaccination, how much do you trust: science, the Standing Committee on Vaccination (STIKO), physicians, the Federal Ministry of Health (BMG), the Federal Centre for Health Education (BZgA)?	1 = no trust at all; 2 = little trust; 3 = moderate trust; 4 = high trust; 5 = very high trust
B. Healthcare workers in paediatric practices	
Vaccination consultations	
Do you conduct HPV vaccination consultations? Do you talk with patients about HPV vaccination?	Yes; No
Attitude towards HPV vaccination^e^	
Vaccinating girls (boys) against HPV is…	1 = very unimportant; 2 = unimportant; 3 = neither unimportant nor important; 4 = important; 5 = very important 1 = very bad; 2 = bad; 3 = neither bad nor good; 4 = good; 5 = very good
Recommendation routine: urgency^f^	
As soon as a girl (a boy) of vaccination age (i.e. 9–14 years) visits the practice, I recommend that the parents have the HPV vaccination carried out immediately.	1 = strongly disagree; 2 = rather disagree; 3 = neither disagree nor agree; 4 = rather agree; 5 = fully agree
Recommendation routine: timeliness	
I routinely recommend HPV vaccination for girls (boys) starting at…	9 years; 10 years; 11 years; 12 years; 13 years; 14 years; 15 years or later
Recommendation frequency	
Thinking about the care of 9–14-year-old patients in your practice: How often do you recommend HPV vaccination to these patients during a visit, if they are not yet vaccinated?	1 = never; 2 = rarely; 3 = occasionally; 4 = often; 5 = always
Experienced parental reactions to the HPV vaccination recommendation^g^	
Please think about the past few months in which you recommended HPV vaccination to parents of 9–14-year-olds. Please indicate how often parents reacted in the following ways to your HPV vaccination recommendation.	1 = never; 2 = rarely; 3 = occasionally; 4 = often; 5 = always
Self-efficacy	
• I feel confident advising parents about the benefits of HPV vaccination • I feel confident advising parents about possible side effects of HPV vaccination • I feel confident educating parents about the sexual transmission of HPV • I feel confident talking about HPV vaccination with parents who are very hesitant • I feel adequately trained to talk about HPV vaccination with hesitant parents • I have enough time during visits to ask parents about their reasons for declining HPV vaccination	1 = strongly disagree; 2 = rather disagree; 3 = neither disagree nor agree; 4 = rather agree; 5 = fully agree
Perceived influence	
• I have influence on parents’ final decision about HPV vaccination • I can convince hesitant parents to have their child vaccinated against HPV	1 = strongly disagree; 2 = rather disagree; 3 = neither disagree nor agree; 4 = rather agree; 5 = fully agree
Knowledge about HPV and HPV vaccination^h, i^	
In your opinion, does the statement apply, does not apply, or do you not know?	Apply; Does not apply; Do not know

HPV: human papillomavirus.

^a^ Subjective norms were calculated by multiplying each normative belief by the motivation to comply. Responses coded as 3 (not applicable/do not know) were considered as missing (NA). If either component was missing, the resulting variable was set to missing. −10: strong subjective norms against vaccination; 10: strong subjective norms in favour of vaccination.

^b^ Correct answers were coded as 1, whereas incorrect and do not know were coded as 0. Missing values were not counted as correct answers and therefore did not contribute to the sum score. 0: no item answered correctly; 9: all items answered correctly.

^c^ For the statements, please see Table 3.

^d^ Perceived relative effectiveness was calculated as the rating of the perceived most effective option minus the rating of HPV vaccination. A value of 0 indicated that HPV vaccination was rated as equally effective as the perceived most effective alternative prevention option.

^e^ Because of low reliability (Spearman–Brown coefficient < 0.60), only one item (unimportant/important) was used for the analysis.

^f^ A Recommendation Quality Index was constructed as the sum of attitude towards HPV vaccination (1–5; higher: more positive), urgency (1–5; higher: greater urgency) and timeliness of recommendation (age category recoded to 0–6; age 9 years: 6 points, age ≥ 15 years: 0 points). 3: low recommendation quality; 16: high recommendation quality.

^g^ For the response options, please see Figure 2.

^h^ Correct answers were coded as 1, whereas incorrect and do not know answers were coded as 0. Missing values were not counted as correct answers and therefore did not contribute to the sum score. 0: no item answered correctly; 6: all items answered correctly.

^I^ For the statements, please see Table 4.

Both questionnaires were pretested (parents: n = 150; HCWs: n = 61) for comprehensibility, filter logic, duration and other quality criteria. The pretest did not lead to substantial revisions.

### Weighting

For the parent survey, a dual-frame weighting and an adjustment weighting were applied. Dual-frame (design) weighting is corrected for differing selection probabilities of the target persons, such as the number of landline and mobile phone numbers available to a respondent. Adjustment weighting accounted for non-response and other biases by aligning selected sample characteristics (in this case: region) with population distributions. For the survey of HCWs, only adjustment weighting (for region) was applied.

### Data analysis

Data were analysed using R (RStudio version 2025.05.1 + 513) (https://www.r-project.org/). Weighted variables were used in the analyses of the responses. Group differences were tested using inferential statistics (t test, ANOVA, α = 0.05).

## Results

Between 13 September and 7 December 2023, we conducted 1,508 interviews with parents. Average duration of the interviews was 25 min. The overall response rate was 22.5% (combined response rate, calculated according to the AAPOR 3 guidelines [25]). Between 12 September and 14 November 2023, 503 interviews were conducted with HCWs in paediatric practices, with an average duration of the interviews of 22 min.

### Parents or guardians

The interviewed parents were an average age of 46.8 years (Table 2). Most of them were women (n = 914; 60.6%), 592 (39.3%) were men and two were non-binary (0.1%). Respondents had an average of 1.4 children aged 9–14 years with a mean age of 11.6 years; 751 (49.8%) were females and 757 (50.2%) were males. Most of these children and adolescents (n = 1,275; 84.7%) visited medical practices with paediatricians. Supplementary Table S3 provides a comparison with official reference data from Germany.

**Table 2:** Characteristics of parents or guardians (n = 1,508) of a child aged 9–14 years and healthcare workers (n = 503) interviewed about human papillomavirus, Germany, 2023

Characteristic	Weighted data^a^		Unweighted data	
	n	%	n	%
A. Parents or guardians (n = 1,508)^b^				
Age (years)				
Mean (SD)	46.7 (6.1)		46.8 (6.0)	
Gender				
Women	897	59.5	914	60.6
Men	609	40.4	592	39.3
Non-binary	2	0.1	2	0.1
Region				
Western federal states (including Berlin)	1,282	85	1,361	90.3
Eastern federal states	226	15	147	9.7
Education (n = 1,505)^c^				
Low	30	2	27	1.8
Intermediate	358	23.8	361	24
High	1,118	74.3	1,117	74.2
Migration history^d^				
Yes	343	22.8	350	23.3
No	1,163	77.2	1,155	76.7
Child (n = 1,508)				
Mean number of children (SD) per parent or guardian	1.4 (0.5)		1.4 (0.5)	
Sex				
Female	743	49.3	751	49.8
Male	765	50.7	757	50.2
Age				
Mean (SD)	11.6 (1.7)		11.6 (1.7)	
Physician of the child (n = 1,506)				
Paediatrician	1,251	83.1	1,275	84.7
General practitioner	235	15.6	207	13.7
Gynaecologist	4	0.3	7	0.5
Other	15	1	17	1.1
B. Healthcare workers in paediatric practices (n = 503)				
Age (in years)				
Mean (SD)	47.5 (12.0)		47.6 (12.2)	
Gender				
Women	349	78.4	393	78.1
Men	108	21.5	109	21.7
Non-binary	1	0.2	1	0.2
Practice location				
Western federal states (including Berlin)	434	86.4	425	84.5
Eastern federal states	69	13.6	78	15.5
Focus of the practice				
Paediatrics	470	93.4	470	93.4
General practice with paediatric care	33	6.6	33	6.6
Profession				
Specialist in paediatrics	256	50.9	261	51.9
Specialist in general practice	8	1.6	9	1.8
Resident in paediatrics	7	1.5	8	1.6
Resident in general practice	1	0.2	1	0.2
Medical assistant	206	40.9	198	39.4
Nurse	25	5.0	26	5.2

SD: standard deviation.

^a^ For the parent survey, a dual-frame weighting and an adjustment weighting were applied. Dual-frame (design) weighting is corrected for differing selection probabilities of the target persons, such as the number of landline and mobile phone numbers available to a respondent. Adjustment weighting accounted for non-response and other biases by aligning selected sample characteristics (in this case: region) with population distributions. For the survey of HCWs, only adjustment weighting (for region) was applied.

^b^ Bremen: n = 303; Bavaria: n = 300.

^c^ Education was classified based on participants’ highest school-leaving qualification and vocational or tertiary qualification using a CASMIN-based classification approach [26]. School-leaving and post-school qualifications were combined to derive three educational levels (low, intermediate, high). Low education comprised participants with no school-leaving qualification or lower secondary education without further qualification; intermediate education comprised participants with vocational qualifications or upper secondary qualifications without tertiary education; and high education comprised participants with tertiary education or upper secondary qualifications combined with vocational qualifications.

^d^ Migration history was defined as the respondent or at least one parent being born outside Germany.

#### Decisions and intentions to vaccinate

Of the interviewed parents, 79% decided jointly about HPV vaccination. In 19%, the mother made the decision, and in 1.5%, the father (0.5%: e.g. parent indicated that their child decided). At the interview moment, the 9–14-year-old child of 34.5% parents was already vaccinated against HPV. Of the remaining parents, 24.8% would definitely vaccinate, 35.2% were undecided to some extent, and 5.6% did not want their child or adolescent to be vaccinated at all (Figure 1).

**Figure 1:**
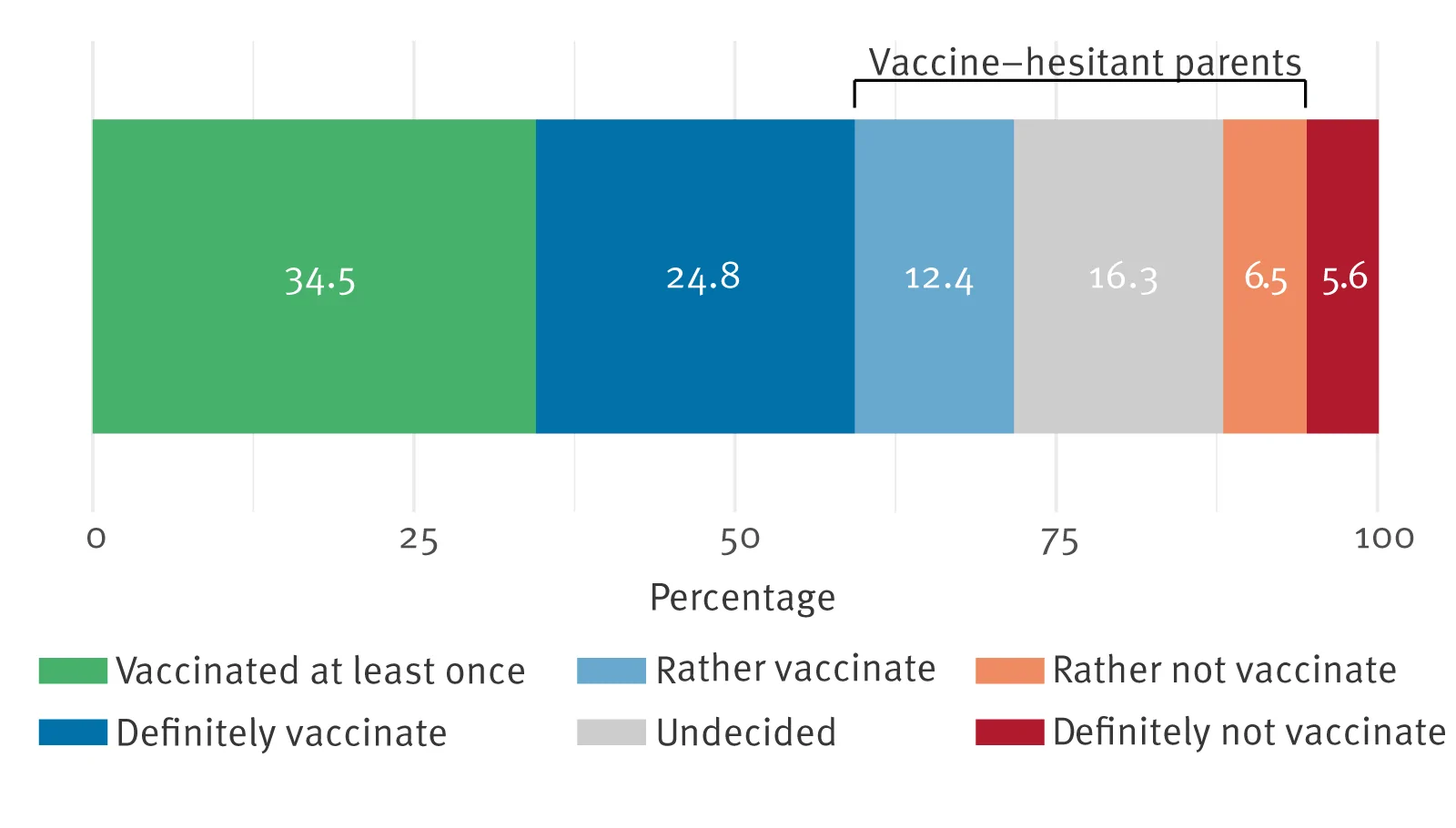
Status and intention of vaccination against human papillomavirus of 9–14-year-olds among parents or guardians, Germany, 2023 (n = 1,508)^a^

^a^ A dual-frame weighting and an adjustment weighting were applied. Dual-frame (design) weighting is corrected for differing selection probabilities of the target persons, such as the number of landline and mobile phone numbers available to a respondent. Adjustment weighting accounted for non-response and other biases by aligning selected sample characteristics (in this case: region) with population distributions.

HPV-specific vaccination hesitancy was operationalised based on parents’ reported intention regarding their child’s HPV vaccination. Following the SAGE Working Group’s conceptualisation of vaccine hesitancy as a continuum between full acceptance and complete refusal [6], parents who were neither fully accepting nor fully refusing HPV vaccination were classified as HPV vaccination-hesitant; this applied to 35.2% of parents.

Parents of unvaccinated daughters were more willing to vaccinate their child (mean (M): 3.9; standard deviation (SD) = 1.3) than those of unvaccinated sons (M: 3.5; SD: 1.3; *t* = −4.7; p < 0.001). Parents with a high level of education (i.e. tertiary education or upper secondary qualification combined with vocational qualification) were more willing to vaccinate their child (M: 3.8; SD: 1.3) than those with an intermediate level of education (i.e. vocational qualification or upper secondary qualification without tertiary education) (M: 3.4; SD: 1.3; *t* = −4.0; p < 0.001) [26]. Vaccination intention was slightly lower among parents with a migration history, i.e. at least one parent or guardian born outside Germany (M: 3.5; SD: 1.3) than among those without a migration history (M: 3.7; SD: 1.3; *t* = −2.0; p = 0.046). Fathers indicated lower vaccination intention (M: 3.4, SD: 1.3) than mothers (M: 3.9; SD: 1.3; *t* = 4.7; p < 0.001). Among parents of a vaccinated child, 94.1% with daughters and 94.5% with sons would vaccinate again, with no significant differences between the sex (M_sons_: 4.7; SD: 0.7; M_daughters_: 4.8; SD: 0.7; *t* = 0.9, p = 0.357).

#### Attitude towards and recommendations for vaccination against human papillomavirus

Of the interviewed parents, 73.6% considered HPV vaccination important for their daughters and 61.4% for their sons (parents of daughters: M: 4.1; SD: 0.9 and those with sons: M: 3.9; SD: 1.0; *t* = −5.8; p < 0.001). Parents of a vaccinated child or adolescent had a more positive opinion on HPV vaccination (M: 4.5; SD: 0.6) than those of an unvaccinated child or adolescent (M: 3.7; SD: 1.0; *t* = −17.6; p < 0.001).

Fewer than half of the parents (43.7%) recalled receiving an active recommendation for HPV vaccination from their HCWs, 21.6% could not remember receiving one, and 34.7% reported not receiving any recommendation. Receiving recommendations was positively associated with the HPV vaccination status (*r* = 0.3; 95% confidence interval (CI): 0.3 to 0.4).

#### Knowledge about human papillomavirus and vaccination against it

Most parents (81.2%) knew about the vaccination recommendation for 9–14-year-olds (Table 3). The most common misconceptions were (i) condom use protects from HPV and (ii) HPV vaccination protects solely against cervical cancer. Parents of daughters (M: 5.6; SD: 2.2) scored lower than those of sons (M: 6.2; SD: 2.3; *t* = 5.1; p < 0.001).

**Table 3:** Knowledge about human papillomavirus and vaccination against it among parents or guardians of 9–14-year-olds, Germany, 2023 (n = 1,508)^a^

Statement	Correctness	Correct answer (%)	Incorrect answer (%)	Do not know (%)
In Germany, HPV vaccination is recommended for 9–14-year-olds	T	81.2	1.6	17.2
My children do not need the vaccination if they are already sexually active	F	77.5	5.7	16.8
HPV vaccination replaces cervical cancer screenings	F	76.6	1.4	22.0
HPV is sexually transmitted	T	75.7	5.4	18.9
HPV infection is always noticeable	F	72.1	2.0	25.9
Only women can have cancer after an HPV infection	F	62.3	10.6	27.0
HPV vaccination protects exclusively against cervical cancer	F	51.2	18.6	30.2
Condoms provide complete protection against HPV	F	49.3	19.9	30.8
HPV vaccination has no negative effects on fertility	T	43.2	13.6	43.2

F: false statement; HPV: human papillomavirus; T: correct statement.

^a^ A dual-frame weighting and an adjustment weighting were applied. Dual-frame (design) weighting is corrected for differing selection probabilities of the target persons, such as the number of landline and mobile phone numbers available to a respondent. Adjustment weighting accounted for non-response and other biases by aligning selected sample characteristics (in this case: region) with population distributions.

#### Perceived effectiveness of prevention measures against human papillomavirus

Respondents were asked to assess the effectiveness of HPV vaccination and other measures for prevention of cervical cancer. Vaccination (M: 4.1; SD: 0.8) and cervical cancer screening (M: 4.1; SD: 1.0) were considered more effective than condom use (M: 3.6; SD: 1.0) or a healthy lifestyle such as non-smoking (M: 3.6; SD: 1.3) (all p < 0.001). Still, 58.6% considered a healthy lifestyle at least somewhat effective, as presented in Supplementary Figure S1. The mean relative perceived effectiveness score for the HPV vaccination was −0.4 (SD: 1.0), indicating that the vaccination was rated as equally effective as the perceived most effective alternative preventive option. In total, 39.8% thought HPV vaccination was less effective than at least one other preventive measure (relative value < 0).

#### Trust

Parents were asked about trust in science, physicians and national authorities involved in HPV vaccination. Science was ranked highest (M: 3.9; SD:  0.8), followed by physicians (M: 3.7; SD: 0.7), STIKO (M: 3.7; SD: 1.0) and the Federal Centre for Health Education (M: 3.6; SD: 0.8). The Federal Ministry of Health (M: 3.3; SD: 1.0) was rated as the least trustworthy compared with all the other options (p < 0.001). The average level of trust did not differ by the sex of their child, but by the vaccination status. Parents who had vaccinated their child (34.5%) against HPV expressed higher trust (M: 3.8; SD: 0.6) than those with an unvaccinated child (M: 3.5; SD: 0.7; *t* = −6.5; p < 0.001).

#### Subjective norms

To determine subjective norms, parents were first asked about the opinions of people in their social environment about their child receiving HPV vaccine and then about the extent to which they intended to follow these opinions, as presented in Supplementary Figure S2. Influence in favour of vaccination was strongest from paediatricians (M: 6.1; SD: 3.6), followed by partners (M: 4.1; SD: 5.1). Relatives (M: 2.0; SD: 3.1), friends (M: 1.9; SD: 3.0) and their own children (M: 2.0; SD: 4.2) exerted less social influence.

#### Parental self-efficacy

Parents were asked about their confidence in their own communication skills about HPV vaccination and their ability to handle information. On average, respondents reported high self-efficacy, especially in discussing vaccination with partners (M: 4.6; SD: 0.7) and paediatricians (M: 4.5; SD: 0.7). The lowest self-efficacy expectations were in finding (M: 4.2; SD: 0.8) and understanding reliable information about HPV vaccination (M: 4.3; SD: 0.8). Mothers reported higher self-efficacy than fathers in discussions with paediatricians (p < 0.001) and children and adolescents (M_mothers_: 4.5; SD: 0.7; M_fathers_: 4.2; SD: 0.8; *t* = −6.5, p < 0.001).

### Survey of healthcare workers in paediatric practices

The 503 respondents were a mean age of 47.6 years, and 393 (78.1%) were women and 109 (21.7%) were men (Table 2). At the time of the survey, 470 (93.4%) worked in paediatric practices and 33 (6.6%) in general practices offering paediatric care. Of all respondents, 279 (55.5%) were physicians (specialists or residents in paediatrics or general practice), 198 (39.4%) were medical assistants and 26 (5.2%) were nurses. Hereafter, medical assistants and nurses are referred to as other HCWs.

Almost all physicians (99.3%) and other HCWs (94.4%) discussed HPV vaccinations in their practices with parents. In 75.8% of cases, the physician initiated the discussion, in 21.1% the other HCWs and in 3.1% the parents.

#### Attitudes towards and recommendations for vaccination against human papillomavirus

Virtually all respondents expressed a (somewhat) positive attitude towards HPV vaccination for girls (99.6%) and boys (98.3%). Attitudes were more positive for vaccination of girls (M: 4.9; SD: 0.3) than of boys (M: 4.9; SD: 0.4; M_diff_: 0.1; 95% CI: 0.1 to 0.1; *t* = 4.5; p < 0.001; *d* = 0.2).

We also asked about the frequency, urgency and timeliness of recommendations. Most HCWs always (61.6%) or often (35.2%) recommended HPV vaccination to unvaccinated children and adolescents. Recommendations were given to 89.8% of girls and 89.4% of boys at the first opportunity. On average, HPV vaccination was recommended for unvaccinated children aged 10 years (SD: 1.3), with girls receiving the recommendation about 0.5 month earlier (M: 10.0; SD: 1.3) than boys (M: 10.0; SD: 1.4; M_diff_: −0.1; 95% CI: −0.1 to −0.0; *t* = −3.6; p = 0.004; *d* = −0.2). For 20.9% of girls and 22.2% of boys, vaccination was recommended only from the age of 12 years onwards.

An index based on attitudes towards HPV vaccination, urgency with which vaccination is recommended and age-appropriate recommendation captured recommendation quality [21,22]. The index was slightly higher for girls (M: 14.5; SD: 1.8) than for boys (M: 14.3; SD: 2.0; M_diff_: 0.2; 95% CI: 0.1 to 0.2; *t* = 5.4; p < 0.001; *d* = 0.2).

#### Perceived parental reactions to vaccination recommendation

We also asked HCWs to rate how often parents exhibited specific reactions to the vaccination recommendations. They reported more frequent acceptance of the HPV vaccination (M: 3.2; SD: 1.0) than a wish to postpone the vaccination to a later visit (M: 3.0; SD: 0.8), as well as parental surprise that the vaccination is due or expressions of concern (Figure 2). Nevertheless, 7.3% of respondents said that parents often or always felt overwhelmed by the vaccination decision, 3.9% encountered rejection and 0.2% had experienced anger as a parental reaction.

**Figure 2:**
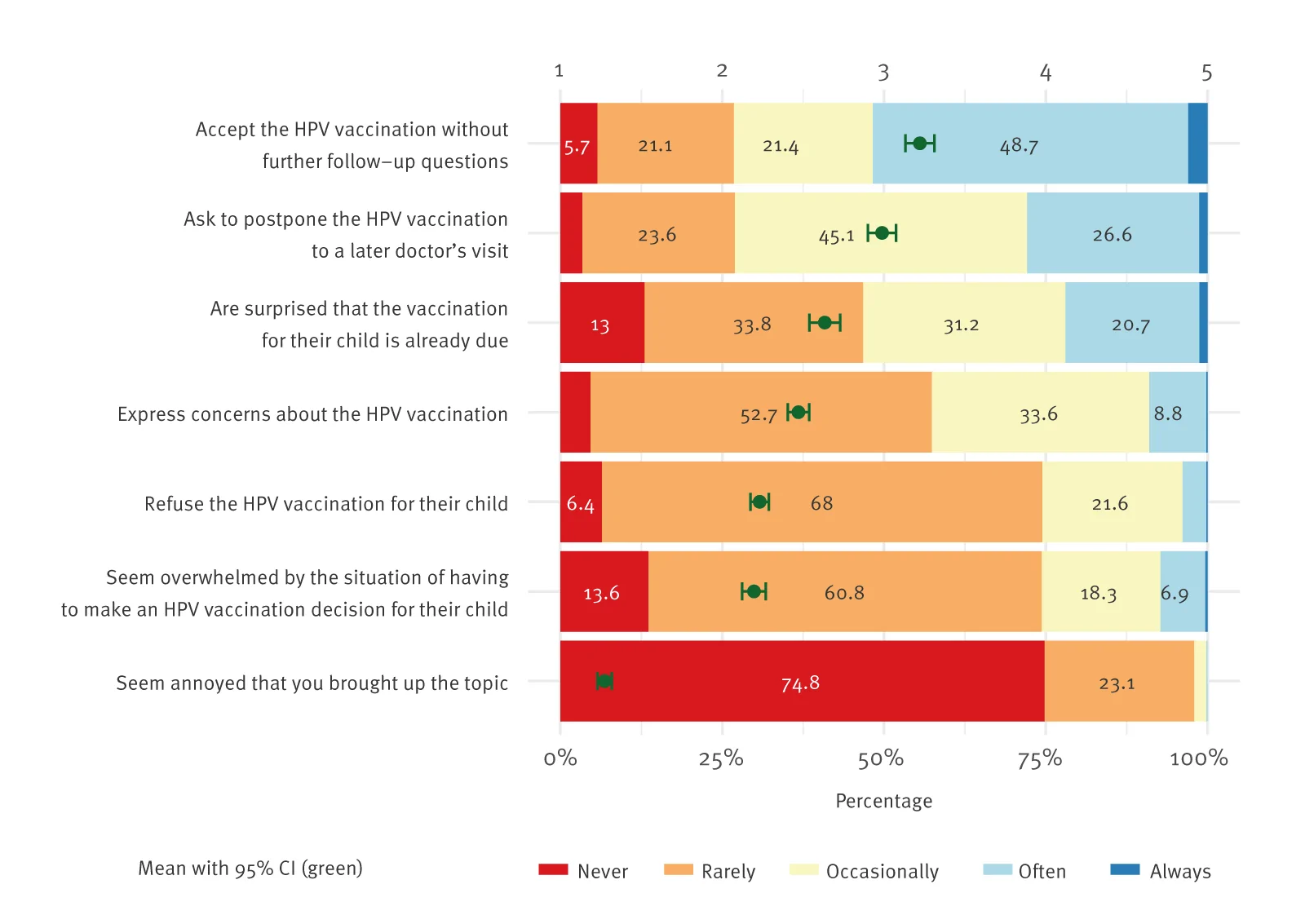
Healthcare workers’ observations of reactions of parents or guardians to the recommendation for vaccination against human papillomavirus, Germany, 2023 (n = 503)^a^

CI: confidence interval; HPV: human papillomavirus.

^a^ Results were weighted using adjustment weighting.

#### Knowledge about human papillomavirus and vaccination against it

Among the knowledge items, questions related to cancer strain coverage and pre-vaccination testing had most correct responses. The most common misconception among HCWs concerned the age-dependent effectiveness of the vaccine. For three of six items, more than a third of participants answered ‘do not know’ (Table 4). Other HCWs gave more uncertain responses to all items. On average, physicians answered 4.2 of six items correctly (SD: 1.2), compared with 3.0 among other HCWs (SD: 1.3; *t* = 10.3; p < 0.001).

**Table 4:** Knowledge about human papillomavirus and vaccination against it among healthcare workers in paediatric practices, Germany, 2023 (n = 503)^a^

Statement	Correctness	Professional group^b^	Correct answer (%)	Incorrect answer (%)	Do not know (%)
9-valent HPV vaccine covers ca 85% of cancer-causing strains	T	Physician	79.5	2.9	17.6
		Other HCW	71.8	0.4	27.8
		Total	76.0	1.8	22.3
Sexually active individuals should be tested for HPV before vaccination	F	Physician	79.1	9.8	11.2
		Other HCW	59.2	16.2	24.7
		Total	70.0	12.7	17.4
HPV types 6 and 11 can cause genital warts	T	Physician	68.3	2.0	29.8
		Other HCW	44.0	2.3	53.7
		Total	57.1	2.1	40.7
Effectiveness of HPV vaccination depends on age of the vaccinee	T	Physician	66.9	23.2	9.8
		Other HCW	41.0	42.6	16.4
		Total	55.0	32.1	12.9
Lifetime risk of genital HPV infection is 70–90%	T	Physicians	57.0	9.3	33.7
		Other HCW	52.0	3.8	44.2
		Total	54.7	6.8	38.5
Individuals diagnosed with HPV should not be vaccinated against HPV	F	Physicians	69.0	13.1	17.9
		Other HCW	35.9	12.3	51.7
		Total	53.8	12.8	33.4

F: false statement; HCW: healthcare worker; HPV: human papillomavirus; T: correct statement.

^a^ Results were weighted using adjustment weighting.

^b^ Physicians: n = 279; other HCWs: n = 224.

Correct answers were based on the national information materials for medical professionals in Germany provided by the Robert Koch Institute and the German National Reference Centre for Papilloma- and Polyomaviruses [46].

#### Self-efficacy

Overall self-efficacy among HCWs was high (M: 4.5; SD: 0.5) but slightly lower on advice on possible side effects (M: 4.4, SD:  0.8). Physicians (M: 4.6, SD = 0.4) rated themselves as more self-efficacious than the other HCWs did (M: 4.4; SD: 0.5; M_diff_: −0.2, 95% CI: −0.2 to −0.1; *t* = −3.1, p = 0.002).

#### Perceived influence on vaccination decisions of parents or guardians

About half of the respondents (51%) felt they could (rather) influence final vaccination decisions of parents (M: 3.5; SD: 1.0). Most (56.9%) considered that they could convince hesitant parents to vaccinate their 9–14-year-old (M: 3.7; SD: 0.7). Physicians were more convinced of their influence on parents (M: 3.7, SD: 0.8) than the other HCWs (M: 3.2, SD: 1.0; M_diff_:  −0.6; 95% CI: −0.7 to −0.4; *t* = −6.6; p < 0.001). Also, the physicians were more confident in convincing hesitant parents to vaccinate (M_physicians_: 3.7, SD:  0.7 vs M_other HCWs_: 3.6, SD: 0.8; M_diff_: −0.2; 95% CI: −0.3 to −0.0; *t* = −2.3; p = 0.022).

## Discussion

In our study, 34.5% of parents with a 9–14-year-old child had vaccinated their child against HPV, and only 5.6% would not vaccinate. These findings are consistent with prior surveys of HPV vaccination intention [27]. However, 35.2% of the interviewed parents were hesitant towards vaccination against HPV. Vaccine hesitancy varies and can be associated with postponing vaccination [6], a behaviour also reported in our survey by HCWs as a common parental reaction to HPV vaccination recommendations. Unlike parents who decline HPV vaccination, parents uncertain about HPV vaccination could be motivated through targeted communication and education [28-30].

Parents of daughters expressed more positive attitudes towards vaccination and were more willing to vaccinate than those of sons. Healthcare workers also considered HPV vaccination of males as slightly less important. This is mirrored in the somewhat lower recommendation quality of HCWs and lower coverage among 15-year-old males in Germany [5]. Although physicians and other HCWs stated that they generally recommended vaccination from the age of 9 years, outpatient billing data for individuals with statutory health insurance show vaccination is often delayed, with over half of the 15-year-olds receiving their first dose at the age of 12–14 years (RKI vaccination surveillance 2023, unpublished data). As the children of the interviewed parents were on average 11.6 years old, a considerable proportion might still be vaccinated later, which is also reflected on the 24.8% of parents reporting a high vaccination intention. This also illustrates that a generally positive attitude towards HPV vaccination does not necessarily translate into vaccination intention. Rather, vaccination decisions are shaped by multiple psychological and contextual factors, including subjective norms, knowledge, trust, self-efficacy and recommendations from healthcare professionals [31].

Addressing these gaps and ensuring full vaccination against HPV requires more systematic education of parents, guardians and HCWs. Communication about HPV vaccination should emphasise the equal importance of female and male vaccination. Equally high coverage for both is achievable as demonstrated in Denmark or Canada [32]. To date, there have been few studies in Germany on the role of mothers in making decisions on vaccinations. Our findings confirm the importance of mothers in HPV vaccination decisions, underscoring them as a key target group for communication [22,33]. While physicians are legally required to give vaccination advice [34], our study shows that other HCWs are also important in addressing parents in Germany. This result highlights the need to specifically include them in training programmes.

Parents have basic knowledge about HPV and HPV vaccination, but notable uncertainties persist, as reported in earlier studies [35]. Gaps are particularly evident in awareness of the consequences of HPV infection and effective protective measures, factors central to risk perception and health behaviour [36]. Misconceptions, such as the strong protective value of condom use or a healthy lifestyle to prevent cervical cancer, illustrate the need for further education. Interestingly, parents of daughters reported lower knowledge scores than those of sons. This might be due to the vaccination having long been referred to as a vaccination against cervical cancer which could have signalled that no further engagement with the topic was necessary. Furthermore, parents may have based their decision on the recommendation of HCWs or their general trust in the healthcare system rather than on actively seeking detailed information, for example from official public health sources in Germany. Prior studies suggest that factual knowledge alone has only limited influence on vaccination decisions [37], and intensive information search may even be associated with a lower vaccination intention [38]. Thus, who conveys information may be as critical as what is conveyed. Notably, our data reveal knowledge gaps and uncertainties, especially among other HCWs, underlining the need for targeted training.

Beyond pure factual knowledge, self-efficacy is another important factor. Since parents and guardians in Germany make health decisions for their children and adolescents until they reach the age of 16 years [39], their confidence in their own communication and information skills is of crucial importance. Parents reported high confidence in discussing HPV vaccination in general and felt self-efficacious in discussing HPV vaccination with their paediatricians. Also, HCWs felt self-efficacious in providing information about HPV. This mutual confidence is a strong foundation for active physician–parent communication. Moreover, paediatricians are a key normative reference for parents, and HCWs considered they could positively influence parental vaccination decisions. Consistent with previous research, our data showed an association between physician recommendation and vaccination status [14,15,40]. However, less than half of the parents recalled receiving such recommendations, highlighting the need for explicit, clear and proactive recommendations. To maintain self-efficacy, especially in conversations with parents and guardians uncertain about HPV vaccination, HCWs need support. Besides closing HPV knowledge gaps, motivational interviewing may be a useful approach, as it strengthens the individual’s motivation to change behaviour [41] while building and maintaining trust between the patient and the provider [28].

The study design entails several methodological limitations. Although telephone surveys are a valuable method for obtaining structured population-based data, sample bias cannot be ruled out. Certain groups (e.g. non-German speakers) are often underrepresented, and differences in telephone coverage and availability may affect participation [42]. To address this, we used a dual-frame approach with 40% mobile phone numbers and weighted the data. However, weighting cannot fully correct all forms of selection bias. Selection bias may also have arisen from varying willingness to participate — essentially, from non-response — as health-interested individuals and those with a positive attitude towards the Robert Koch Institute may have been more likely to participate [43]. In addition, socially desirable responding cannot be excluded. To mitigate this effect, anonymity was ensured, all opinions were treated as valuable, interviews were standardised and interviewers were trained accordingly.

The sample of 503 HCWs enabled analyses of physicians and other HCWs but did not allow stratification. Still, as a telephone survey based on a broad sampling of paediatric practices, it provides a comparatively large and robust database. The HCWs’ assessments refer only to parents and guardians who visit their practices. Families without regular paediatric care – an increasing number in Germany [44,45] – could not be captured. A key strength of the study is surveying parents and HCWs on the same topic. The surveys also had clear practical public health relevance, as they were designed to guide training programmes and were based on validated items. In addition, the findings were translated into public health practice in several ways. For example, key messages were developed and incorporated into the national communication strategy for HPV vaccination and identified knowledge gaps were addressed through new frequently asked questions on the website of the Robert Koch Institute and corresponding information in its materials for HCWs.

## Conclusion

Although HPV vaccination has been recommended for all children and adolescents in Germany since 2018, parents and HCWs still consider HPV vaccination more important for females than for males. Reducing these gender/sex specific perceptions is important to promote equitable HPV vaccination uptake. Healthcare workers are crucial in informing and supporting parents and guardians, particularly those who are vaccine hesitant. Physicians should be further supported in this role, and medical assistants and nurses should be more actively involved in HPV vaccination communication. This is particularly important, as fewer than half of parents recalled receiving a vaccination recommendation. Public health strategies should therefore promote consistent, gender-neutral HPV communication and strengthen communication skills across entire healthcare teams.

## Data Availability

The datasets analysed are available from the corresponding author upon reasonable request. Researchers wishing to request access to the data may contact the corresponding author. Access is restricted to qualified researchers and is subject to review by the study team, approval of a written proposal, and completion of a data access agreement.

## Supplementary Data

443000 Supplementary Material
