# Supplementary material for: Understanding vaccination against human papillomavirus (HPV): knowledge and perceptions among parents or guardians and healthcare workers, Germany, 2023
Source: Euro Surveill. 2026 Oct 15;31(41):2500910. doi: 10.2807/1560-7917.ES.2026.31.41.2500910 (PMC13675697; doi:10.2807/1560-7917.ES.2026.31.41.2500910)
Supplement: Supplementary file 1 [file 25-00910_SCHMID-KUPKE_Supplement.pdf]

Supplement

This supplementary material is hosted by Eurosurveillance as supporting information alongside the article **"Understanding vaccination against human papillomavirus (HPV): knowledge and perceptions among parents or guardians and healthcare workerns, Germany, 2023"**, on behalf of the authors, who remain responsible for the accuracy and appropriateness of the content. The same standards for ethics, copyright, attributions and permissions as for the article apply. Supplements are not edited by Eurosurveillance and the journal is not responsible for the maintenance of any links or email addresses provided therein.

Supplementary Table S1 – Measurement table for the parent questionnaire 2

Supplementary Table S2 – Measurement table for the healthcare worker questionnaire 9

Supplementary Table S3 – Description of the study population with comparison to official reference data from Germany 15

Supplementary Figure S1. Perceived effectiveness of measures for the prevention of cervical cancer. 17

Supplementary Figure S2. Normative beliefs and motivation to comply with people in parent's social environment. 17

References 18

Supplementary Table S1 – Measurement table for the parent questionnaire

Measure	Item	Response options	Reference*
**Gender of responding parent**	What gender do you identify with?	Women<br>Men<br>Non-binary<br>No response	
**Age of responding parent**	How old are you?	*Open response*	
Selection of child<br><br>**Age of child**	How many children aged 9-14 years are you the legal guardian of?	*Open response*	
**Sex of selected child**	What sex was entered on your child's birth certificate?	Male<br>Female<br>No response	
**Gender of selected child**	What gender does your child identify with?	Women<br>Men<br>Non-binary<br>No response	
**Child's HPV vaccination status**	Has your child been vaccinated against HPV?	Yes, my child has received two HPV vaccinations<br>Yes, my child has received one HPV vaccination<br>No, my child has not or not yet been vaccinated against HPV<br>I don't remember	
Filter: if vaccinated<br><br>**Timing of vaccination**	*Filter: if vaccinated*<br><br>At what age did your child received the first HPV vaccination?	At age 9<br>At age 10<br>At age 11<br>At age 12<br>At age 13<br>At age 14	
**HPV vaccination intention**	*Filter: if unvaccinated*<br><br>Do you intend to have your child vaccinated against HPV?<br><br>*Filter: if vaccinated*<br><br>Imagine you had to make the decision about HPV vaccination for your child again: Would you have your child vaccinated against HPV?	1 = definitely not vaccinate<br>2 = rather not vaccinate<br>3 = undecided<br>4 = rather vaccinate<br>5 = definitely vaccinate	(1)<br><br>(2)
**Vaccination decision-maker**	Who mainly makes the vaccination decisions for your child?	You yourself<br>The other parent<br>Both parents together<br>Other, namely:	
**Gender of the other parent**	What gender does the other parent identify with?	Women<br>Men<br>Non-binary<br>No response	

Measure	Item	Response options	Reference*
**Attitude towards HPV vaccination**	Vaccinating my child against HPV is…	1 = very unimportant<br>2 = unimportant<br>3 = neither unimportant nor important<br>4 = important<br>5 = very important<br><br>1 = very bad<br>2 = bad<br>3 = neither bad nor good<br>4 = good<br>5 = very good	(1, 2)
**Risk perception**	What do you think – how likely is it that your child will ever develop [*filter for girls:* cervical cancer; *filter for boys:* HPV-related cancer of the mouth and throat]?	1 = very low<br>2 = low<br>3 = moderate<br>4 = high<br>5 = very high	(1-3)
Filter: if unvaccinated<br><br>**Anticipated regret – having declined HPV vaccination (inaction regret)**	*Filter: if unvaccinated*<br><br>Imagine your child never receives the HPV vaccination and at some point in the future develops [*filter for girls:* cervical cancer; *filter for boys:* HPV-related cancer of the mouth and throat].<br>How much would you regret your decision not to have your child vaccinated?	1 = not regret at all<br>2 = regret a little<br>3 = partly<br>4 = somewhat regret<br>5 = strongly regret	(1, 2)
**Anticipated regret – having received HPV vaccination (action regret)**	Imagine [*filter for parents of unvaccinated children:* your child has received the HPV vaccination and] your child experiences a severe side effect.<br>How much would you regret your decision to have your child vaccinated against HPV?	1 = not regret at all<br>2 = regret a little<br>3 = partly<br>4 = somewhat regret<br>5 = strongly regret	(2)
**Decisional conflict regarding HPV vaccination (decisional conflict)**	Please indicate how much you agree or disagree with each statement.<br><br>Regarding the HPV vaccination for my child…	1 = strongly disagree<br>2 = rather disagree<br>3 = neither disagree nor agree<br>4 = rather agree<br>5 = fully agree	(1, 2)
	… I feel confident in my decision for or against the vaccination	*see above*	
	… the decision is easy for me to make		
**Beliefs about HPV vaccination (attitude roots)**	Please again indicate how much you agree or disagree with each statement.	1 = strongly disagree<br>2 = rather disagree<br>3 = neither disagree nor agree<br>4 = rather agree<br>5 = fully agree	(4, 5)
[attitude root: distrust]	The HPV vaccination was only introduced because the pharmaceutical industry wants to make a lot of money from it.	*see above*	(4, 5)

Measure	Item	Response options	Reference*
[attitude root: distrust]	Health authorities, politicians, and governments are corrupt and profit from the HPV vaccination.		(4, 5)
[attitude root: distrust]	Too little is known about whether the HPV vaccination effectively protects against cervical cancer.		(1, 4, 5)
[attitude root: moral concerns]	The HPV vaccination can arouse sexual curiosity and lead to earlier sexual activity.		(4, 5)
[attitude root: moral concerns]	Topics such as HPV and sexuality should not yet be discussed at this age.		(4, 5)
[attitude root: fears and phobia]	Too little is known about the harmful side effects of the HPV vaccination.		(1, 4, 5)
[attitude root: unwarranted beliefs]	In addition to other vaccinations, the HPV vaccination can overload the immune system.		(4, 5)
[attitude roots: unwarranted beliefs]	My child does not need the HPV vaccination because they are not yet sexually active.		(4, 5)
[attitude roots: unwarranted beliefs]	Instead of HPV vaccination, people should rather improve other factors such as good hygiene, a healthy lifestyle, and protective measures against the disease.		(4, 5)
[attitude roots: distorted risk Perception]	My child does not need the HPV vaccination because an HPV infection usually has no consequences.		(4-6)
**Subjective norms regarding HPV vaccination:**<br><br>Normative beliefs	What do you expect – what is the opinion of …<br>• your partner<br>• your daughter/ your son<br>• parents you are friends with<br>• your relatives<br>• your child's paediatrician<br>… regarding HPV vaccination of your child [*for daughter/son:* regarding their own HPV vaccination]?	-2 = definitely not vaccinate<br>-1 = rather not vaccinate<br>0 = undecided<br>1 = rather vaccinate<br>2 = definitely vaccinate<br><br>3 = not applicable / don't know;	(1, 2)
**Subjective norms regarding HPV vaccination:**<br><br>Motivation to comply	How motivated are you to follow the opinion of…<br>*(see above)* ?	1 = not at all motivated<br>2 = rather not motivated<br>3 = neither unmotivated nor motivated<br>4 = rather motivated<br>5 = fully motivated	
**Habit strength regarding HPV vaccination**	Making the decision about HPV vaccination for my child is something I …	1 = strongly disagree<br>2 = rather disagree<br>3 = neither disagree nor agree<br>4 = rather agree<br>5 = fully agree	(1, 2)

Measure	Item	Response options	Reference*
	… do automatically	*see above*	
	… do without thinking		
**Self-efficacy expectations regarding HPV vaccination**	To what extent would you be able to succeed in the following situations:	1 = not at all able to succeed<br>2 = rather not able to succeed<br>3 = partly able to succeed<br>4 = rather able to succeed<br>5 = fully able to succeed	(1)
	Having a good conversation about HPV vaccination with my child	*see above*	
	Having a good conversation about HPV vaccination with my partner		
	Having a good conversation about HPV vaccination with general practitioner		
	Finding reliable information about HPV vaccination;		(2)
	Understanding information about HPV		(2)
**Knowledge about HPV and HPV vaccination**	HPV is sexually transmitted.	Apply<br>Does not apply<br>Do not know	(1)
	HPV infection is always noticeable.	*see above*	
	My child does not need the vaccination if they are already sexually active.		
	Only women can develop cancer from an HPV infection.		
	HPV vaccination protects exclusively against cervical cancer.		
	HPV vaccination has no negative effect on fertility.		(7)
	HPV vaccination replaces later cervical cancer screening.		
	Condoms provide complete protection against HPV.		
	In Germany, HPV vaccination is recommended at ages 9–14.		(2)
**Perceived relative effectiveness of HPV vaccination**	How do you rate the effectiveness of the following options for preventing cervical cancer?	1 = not at all effective<br>2 = rather not effective<br>3 = partly effective<br>4 = rather effective<br>5 = fully effective	(1, 2)
	Using condoms	*see above*	

Measure	Item	Response options	Reference*
	Having sexual intercourse with only one partner in life		
	[for women:] Participation in cervical cancer screening		
	Healthy lifestyle (e.g. not smoking)		
	HPV vaccination		
**Trust in institutions involved in HPV vaccination**	Regarding HPV vaccination, how much do you trust…	1 = no trust at all<br>2 = little trust<br>3 = moderate trust<br>4 = high trust<br>5 = very high trust	(2)
	Science	*see above*	
	The Standing Committee on Vaccination (STIKO)		
	Physicians		
	The Federal Ministry of Health (BMG)		
	The Federal Centre for Health Education (BZgA)		
**Type of medical care**	Which type of physician mainly provides medical care for your child?	General practitioner<br>Paediatrician<br>Gynaecologist<br>Other, namely:	
**Participation in preventive medical check-ups**	Please indicate the last U or J examination your child participated in. If you do not remember, please estimate.	U9, at age 5<br>U10, at age 7-8<br>U11, at age 9-10<br>J1, at age 12-14	
**Physician's recommendation for HPV vaccination**	Did your doctor actively recommend the HPV vaccination for your child on their own initiative?	Yes<br>No<br>I don't remember	
**Socioeconomic status**	What is your highest level of general schooling completed?	• No school-leaving certificate / left school without a certificate or still attending school<br>• Lower secondary school certificate (Hauptschule / Volksschule) or comparable (POS grade 8/9 or up to 7 years of schooling)<br>• Intermediate secondary school certificate (Realschule / Mittlere Reife / POS grade 10 or equivalent)<br>• Higher education entrance qualification (Abitur / subject-specific university entrance qualification / university of applied sciences entrance qualification)	(8)

Measure	Item	Response options	Reference*
	Do you have a vocational qualification or a degree from a university or university of applied sciences?	No<br>Yes, through vocational or school-based training<br>Yes, through a university degree (Note: studies at a university or university of applied sciences)	
	What is your household's total monthly net income? This refers to the total amount from wages, salaries, self-employment, pensions, or retirement income. Please also include public benefits, rental income, housing benefits, child benefits, and any other sources of income. Then deduct taxes, business expenses, and social security contributions.	*Open response*	
	If you cannot specify the exact amount, please assign yourself to one of the following categories:	1 to under 700 EUR<br>700 to under 1,100 EUR<br>1,100 to under 1,500 EUR<br>1,500 to under 2,000 EUR<br>2,000 to under 2,500 EUR<br>2,500 to under 3,500 EUR<br>3,500 to under 4,500 EUR<br>4,500 to under 5,500 EUR<br>5,500 to under 7,500 EUR<br>7,500 to under 10,000 EUR<br>10,000 to under 18,000 EUR<br>18,000 EUR or more	
**Migration background**	In which country were you born?<br>In which country was your mother born?<br>In which country was your father born?	In Germany<br>In another country	
**Place of residence**	In which federal state do you live in?	Baden-Württemberg<br>Bavaria<br>Berlin<br>Brandenburg<br>Bremen<br>Hamburg<br>Hesse<br>Mecklenburg-Western Pomerania<br>Lower Saxony<br>North Rhine-Westphalia<br>Rhineland-Palatinate<br>Saarland<br>Saxony<br>Saxony-Anhalt<br>Schleswig-Holstein<br>Thuringia	
	We would also like to know how many inhabitants live in the place where you reside. If in doubt, please refer to the smaller unit, e.g., the village rather than a larger administrative association.	fewer than 2,000<br>fewer than 5,000<br>fewer than 20,000<br>fewer than 50,000<br>fewer than 100,000<br>fewer than 500,000	

Measure	Item	Response options	Reference*
		500,000 inhabitants and more	
**Mobile phone use**	How many other people aged 18 or older regularly use the mobile phone I am calling you on, besides you?	No one else<br>One<br>Two<br>Three<br>Four<br>Five	
	How many mobile phone numbers are you personally reachable on, i.e., use for phone calls, whether for work or private purposes?	None (landline only)<br>One<br>Two<br>Three<br>Four<br>Five	
	Now about your landline connection: On how many different landline numbers (with your local area code) can you be reached at home? Please do not include numbers that belong exclusively to a fax machine or computer.	None (mobile only)<br>One<br>Two<br>Three<br>Four<br>Five	

* If the items are based on a publication, the source is indicated.

Supplementary Table S2 – Measurement table for the healthcare worker questionnaire

Measure	Item	Response options	Reference*
**Medical specialty**	What is your practice's medical specialty or main area of work?	Paediatrics<br>General medicine with paediatric care<br>Other<br>Don't know<br>No response	
**Gender of respondent**	What gender do you identify with?	Women<br>Men<br>Non-binary<br>No response	(6, 9)
**Age of respondent**	How old are you?	*Open response*	(6)
**Professional title / medical field**	Which professional title applies to you?	Specialist in paediatrics and adolescent medicine<br>Specialist in general medicine<br>Resident in paediatrics and adolescent medicine<br>Resident in general medicine<br>Medical assistant<br>Health and nursing professional / nurse<br>Other, namely:	(6, 9, 10)
**Practice location**	In which federal state is your practice located?	Schleswig-Holstein<br>Hamburg<br>Lower Saxony<br>Bremen<br>North Rhine-Westphalia<br>Hesse<br>Rhineland-Palatinate<br>Baden-Württemberg<br>Bavaria<br>Saarland<br>Berlin<br>Brandenburg<br>Mecklenburg-Western Pomerania<br>Saxony<br>Saxony-Anhalt<br>Thuringia	
**Filter question 1**	Now we would like to ask about conversations in your practice regarding HPV vaccination: Do you conduct HPV vaccination consultations?	Yes<br>No	
	You indicated that you do not conduct HPV vaccination consultations. Please now think about general exchanges with patients: Do you talk with patients about HPV vaccination?	Yes<br>No	
**Initiative for HPV vaccination**	*if Filter question 1 = No:*<br><br>Who usually brings up the topic of HPV vaccination?	You yourself<br>The parent<br>The physician<br>The children or adolescents	

Measure	Item	Response options	Reference*
**discussions/ consultations**			
	Who usually initiates the HPV vaccination consultations?	You yourself<br>The parent<br>The physician<br>The children or adolescents	
**Recommendation frequency**	Thinking about the care of 9–14-year-old patients in your practice: How often do you recommend HPV vaccination to these patients during a visit, if they are not yet vaccinated?	1 = never<br>2 = rarely<br>3 = occasionally<br>4 = often<br>5 = always	(6)
**Attitude towards HPV vaccination**	Vaccinating girls [boys] against HPV is…	1 = very unimportant<br>2 = unimportant<br>3 = neither unimportant nor important<br>4 = important<br>5 = very important<br><br>1 = very bad<br>2 = bad<br>3 = neither bad nor good<br>4 = good<br>5 = very good	(6, 9, 11, 12)
**Risk perception**	What do you think — how high is the chance that an unvaccinated girl will ever develop HPV-related cancer, such as cervical cancer?<br><br>What do you think — how high is the chance that an unvaccinated boy will ever develop HPV-related cancer, such as cancer of the mouth and throat?	1 = very low<br>2 = low<br>3 = moderate<br>4 = high<br>5 = very high	(7)
**Recommendation routine: urgency**	*[only if recommendation frequency ≠ never]*<br><br>As soon as a girl [a boy] of vaccination age (i.e., 9–14 years) visits the practice, I recommend that the parents have the HPV vaccination carried out immediately.	1 = strongly disagree<br>2 = rather disagree<br>3 = neither disagree nor agree<br>4 = rather agree<br>5 = fully agree	(9, 11)
**Recommendation routine: timeliness**	*[only if recommendation frequency ≠ never]*<br><br>I routinely recommend HPV vaccination for girls [boys] starting at …	• 9 years<br>• 10 years<br>• 11 years<br>• 12 years<br>• 13 years<br>• 14 years<br>• 15 years or later	(9)
**Prevention message**	*[only if recommendation frequency ≠ never]*<br><br>What is your main message when informing girls [boys] and their parents about HPV vaccination?	The HPV vaccination prevents cervical cancer.<br>The HPV vaccination prevents not only cervical cancer but also other HPV-related cancers, such as cancers of the mouth and throat.	(11)

Measure	Item	Response options	Reference*
		The HPV vaccination prevents genital warts.<br>The spread of the HPV virus is reduced.<br>The vaccination is recommended by the Standing Committee on Vaccination (STIKO).<br>Other, namely:	
**Experienced parental reactions to the HPV vaccination recommendation**	Please think about the past few months in which you recommended HPV vaccination to parents of 9–14-year-olds.<br><br>Please indicate how often parents reacted in the following ways to your HPV vaccination recommendation:	1 = never<br>2 = rarely<br>3 = occasionally<br>4 = often<br>5 = always	(6)
	The parents ask to postpone the HPV vaccination to a later doctor's visit.	*see above*	(6)
	The parents accept the HPV vaccination without further follow-up questions.		(6)
	The parents express concerns about the HPV vaccination.		(6)
	The parents refuse the HPV vaccination for their child.		(6)
	The parents seem annoyed that you brought up the topic.		(6)
	The parents seem overwhelmed by the situation of having to make an HPV vaccination decision for their child.		
	The parents are surprised that the vaccination for their child is already due.		
**Perceived frequency of parents' attitude roots**	I will now read to you several statements that are often used as arguments against HPV vaccination. All of these statements are false or misleading.<br><br>Please indicate how often you experience parents expressing statements of this kind as reasons for not having their children vaccinated against HPV. Do you experience this…	1 = never<br>2 = rarely<br>3 = occasionally<br>4 = often<br>5 = always	
[attitude root: distrust]	Health authorities, politicians, and governments are corrupt and profit from the HPV vaccination.	*see above*	(4, 5)
[attitude root: distrust]	The HPV vaccination was only introduced because the		(4, 5)

Measure	Item	Response options	Reference*
	pharmaceutical industry wants to make a lot of money from it.		
[attitude root: distrust]	Too little is known about whether the HPV vaccination effectively protects against cervical cancer.		(1, 4, 5)
[attitude root: moral concerns]	The HPV vaccination can arouse sexual curiosity and lead to earlier sexual activity.		(4, 5)
[attitude root: moral concerns]	Topics such as HPV and sexuality should not yet be discussed at this age.		(4, 5)
[attitude root: fears and phobia]	Too little is known about the harmful side effects of the HPV vaccination.		(1, 4, 5)
[attitude root: unwarranted beliefs]	In addition to other vaccinations, the HPV vaccination can overload the immune system.		(4, 5)
[attitude roots: unwarranted beliefs]	My child does not need the HPV vaccination because they are not yet sexually active.		(4, 5)
[attitude roots: unwarranted beliefs]	Instead of HPV vaccination, people should rather improve other factors such as good hygiene, a healthy lifestyle, and protective measures against the disease.		(4-6)
[attitude roots: distorted Risk Perception]	My child does not need the HPV vaccination because an HPV infection usually has no consequences.		(6)
**Perceived difficulty of refuting parents' attitude roots**	I will now read the statements to you again.<br><br>Please indicate, for each statement, how easy or difficult it would be for you to refute it in a conversation with a patient or with parents.<br><br>Items: *see above*	1 = very easy<br>2 = easy<br>3 = undecided<br>4 = difficult<br>5 = very difficult	(4, 5)
**Use of motivational interviewing techniques** with vaccine-hesitant parents	The following questions refer to your communication style.<br><br>Please indicate how often you use the following behaviors when interacting with parents who are hesitant about HPV vaccination. Do you use these…?	1 = never<br>2 = rarely<br>3 = occasionally<br>4 = often<br>5 = always	
	I use open-ended questions to explore parents' concerns.	*see above*	
	I express that I understand the parents' concerns.		

Measure	Item	Response options	Reference*
	I summarize the parents' arguments in my own words.		
	I tell parents that, in their position, I would have my child vaccinated.		
**Self-efficacy**	Please indicate to what extent you personally agree with the following statements.	1 = strongly disagree<br>2 = rather disagree<br>3 = neither disagree nor agree<br>4 = rather agree<br>5 = fully agree	
	I feel confident advising parents about the benefits of HPV vaccination	*see above*	(6, 13)
	I feel confident advising parents about possible side effects of HPV vaccination		(13)
	I feel confident educating parents about the sexual transmission of HPV		(12)
	I feel confident talking about HPV vaccination with parents who are very hesitant		(12, 13)
	I feel adequately trained to talk about HPV vaccination with hesitant parents		(13)
	I have enough time during visits to ask parents about their reasons for declining HPV vaccination		(6)
**Perceived influence**	I have influence on parents' final decision about HPV vaccination	1 = strongly disagree<br>2 = rather disagree<br>3 = neither disagree nor agree<br>4 = rather agree<br>5 = fully agree	(6)
	I can convince hesitant parents to have their child vaccinated against HPV	*see above*	(6)
**Knowledge about HPV and HPV vaccination**	The lifetime risk of genital HPV infection is 70–90%.	Apply<br>Does not apply<br>Do not know	(14)
	HPV types 6 and 11 can cause genital warts.	*see above*	(14, 15)
	The 9-valent HPV vaccine covers about 85% of cancer-causing strains.		(12)
	Sexually active individuals should be tested for HPV before vaccination.		(16)
	Individuals diagnosed with HPV should not be vaccinated against HPV.		(16)

Measure	Item	Response options	Reference*
	The effectiveness of HPV vaccination depends on age.		(12)

* If the items are based on a publication, the source is indicated.

Supplementary Table S3 – Description of the study population with comparison to official reference data from Germany

	Study population: Parents with at least one child aged 9–14		Official reference data from Germany (17)
	Weighted data	Unweighted data	Closest available comparison group
**Age in years**			Reference group: Mothers and fathers with a youngest child under 18 years
Mean (SD)	46.7 (6.1)	46.8 (6.0)	Age groups:<br>< 25: 1.3%;<br>25–35: 22.2%;<br>35–45: 43.6%;<br>45–55: 27.9%;<br>55–65: 4.6%;<br>65–75: 0.3%.
**Gender, n (%)**			Reference group: Mothers and fathers with a youngest child under 18 years
Male	609 (40.4%)	592 (39.3%)	Fathers: 6,990,000 (46.6%)
Female	897 (59.5%)	914 (60.6%)	Mothers: 8,023,000 (53.4%)
Non-binary	2 (0.1%)	2 (0.1%)	No comparable reference category was available for non-binary respondents
**Region, n (%)**			Reference group: Families with children under 18 years
Western federal states (incl. Berlin)	1,282 (85.0%)	1,361 (90.3%)	7,014,000 (85.0%)
Eastern federal states	226 (15.0%)	147 (9.7%)	1,235,000 (15.0%)
**Education, n (%)**			Reference group: Person who provides the main income for the household; not limited to parents and therefore only indirectly comparable.
Low	30 (2.0%)	27 (1.8%)	Lower secondary school-leaving certificate: 27.4%;<br>school-leaving certificate from a polytechnic secondary school: 7.2%;<br>intermediate secondary school-leaving certificate or equivalent: 23.2%;<br>higher education entrance qualification: 38.0%;<br>no general school-leaving qualification: 3.9%.
Intermediate	358 (23.8%)	361 (24.0%)	
High	1,118 (74.3%)	1,117 (74.2%)	
**Number of children**			Reference group: Families with children under 18 years
Mean (SD)	1.4 (0.5)	1.4 (0.5)	

			1 child: 4,133,000 (50.1%)<br>2 children: 3,142,000 (38.1%)<br>3 children: 765,000 (9.3%)<br>4 children: 156,000 (1.9%)<br>≥ 5 children: 54,000 (0.6%)
			Reference group: Families with children under 18 years
**Migration background, n (%)**			
No	1,163 (77.2%)	1,155 (76.7%)	4,897,000 (59.4%)
Yes	343 (22.8%)	350 (23.3%)	3,353,000 (40.6%)

Note: Official reference data refer to the closest available comparison groups and are not always directly comparable with the study population. Age and gender refer to mothers and fathers with a youngest child under 18 years; region, number of children, and migration history refer to families with children under 18 years; education refers to the person providing the main income for the household and is not limited to parents.

Supplementary Figure S1. Perceived effectiveness of measures for the prevention of cervical cancer.

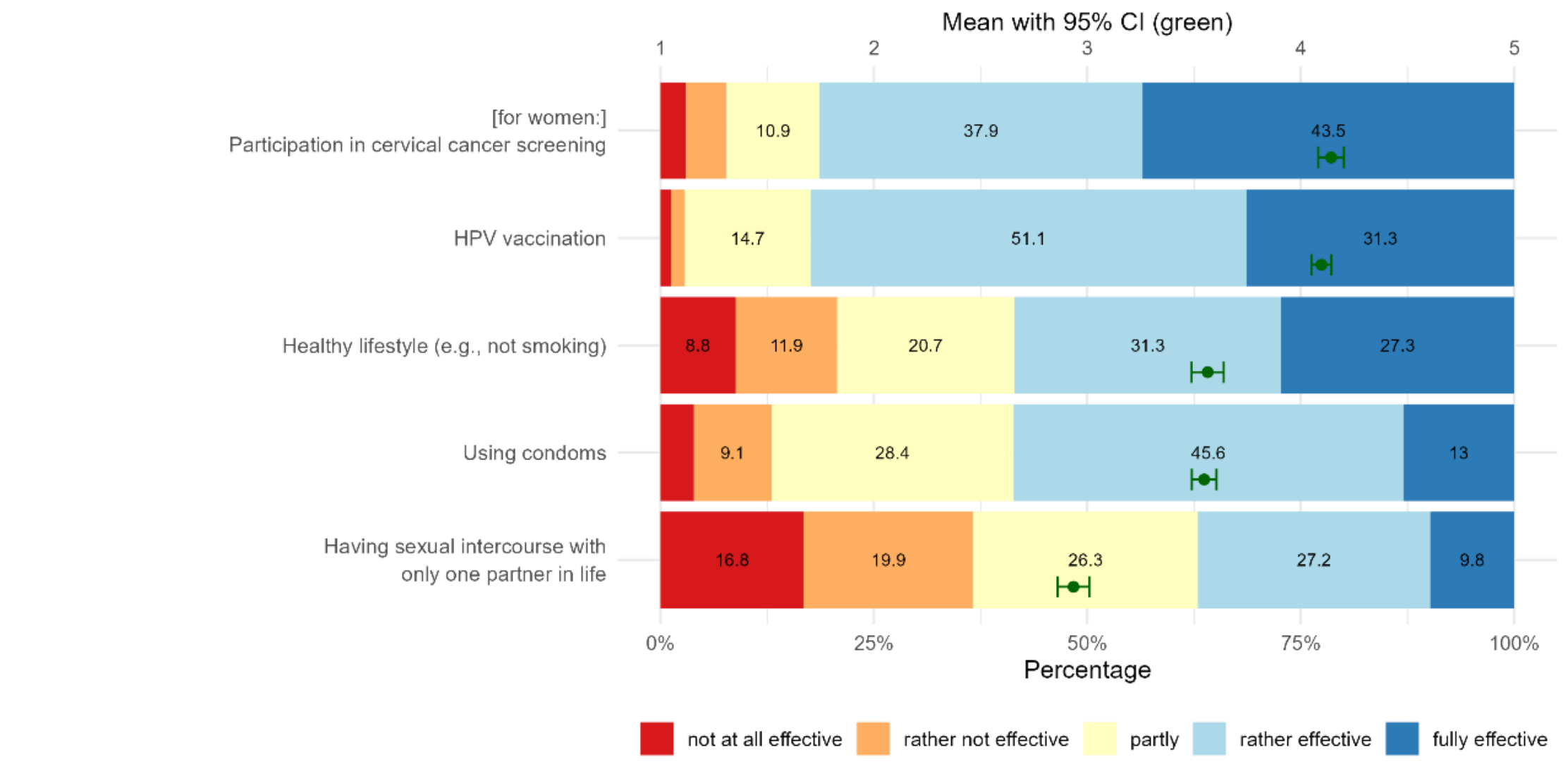

Supplementary Figure S2. Normative beliefs and motivation to comply with people in parent's social environment.

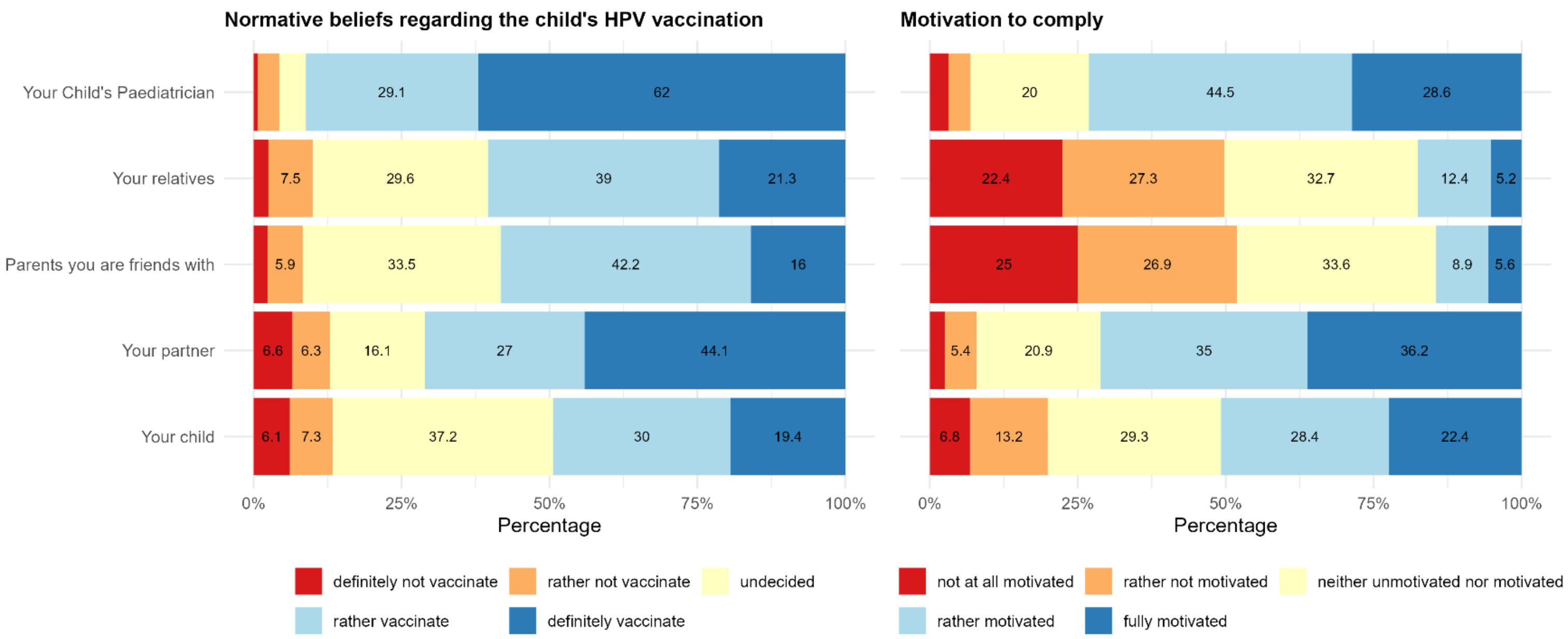

References

1. Pot M, Van Keulen HM, Ruiter RA, Eekhout I, Mollema L, Paulussen TW. Motivational and contextual determinants of HPV-vaccination uptake: A longitudinal study among mothers of girls invited for the HPV-vaccination. Preventive medicine. 2017;100:41–9.
2. van Keulen HM, Otten W, Ruiter RA, Fekkes M, van Steenbergen J, Dusseldorp E, et al. Determinants of HPV vaccination intentions among Dutch girls and their mothers: a cross-sectional study. BMC public health. 2013;13:1–21.
3. Reiter PL, Brewer NT, Gottlieb SL, McRee A-L, Smith JS. Parents' health beliefs and HPV vaccination of their adolescent daughters. Social science & medicine. 2009;69(3):475–80.
4. Fasce A, Schmid P, Holford DL, Bates L, Gurevych I, Lewandowsky S. A taxonomy of anti-vaccination arguments from a systematic literature review and text modelling. Nature human behaviour. 2023;7(9):1462–80.
5. Holford D, Schmid P, Fasce A, Garrison A, Karlsson L, Taubert F, et al. Difficulties faced by physicians from four European countries in rebutting antivaccination arguments: a cross-sectional study. BMJ Public Health. 2024;2(1).
6. McRee A-L, Gilkey MB, Dempsey AF. HPV vaccine hesitancy: findings from a statewide survey of health care providers. Journal of Pediatric Health Care. 2014;28(6):541–9.
7. Karafillakis E, Simas C, Jarrett C, Verger P, Peretti-Watel P, Dib F, et al. HPV vaccination in a context of public mistrust and uncertainty: a systematic literature review of determinants of HPV vaccine hesitancy in Europe. Human vaccines & immunotherapeutics. 2019.
8. Müters S, Michalski N, Hoebel J. Aktualisierung der Berechnungsgrundlagen für den Index des sozioökonomischen Status in der Studie Gesundheit in Deutschland aktuell (GEDA) 2019/2020-EHIS. 2023.
9. Gilkey MB, Malo TL, Shah PD, Hall ME, Brewer NT. Quality of physician communication about human papillomavirus vaccine: findings from a national survey. Cancer Epidemiology, Biomarkers & Prevention. 2015;24(11):1673–9.
10. Brewer NT, Hall ME, Malo TL, Gilkey MB, Quinn B, Lathren C. Announcements versus conversations to improve HPV vaccination coverage: a randomized trial. Pediatrics. 2017;139(1).
11. Gilkey MB, McRee A-L. Provider communication about HPV vaccination: a systematic review. Human vaccines & immunotherapeutics. 2016;12(6):1454–68.
12. Kumar MM, Boies EG, Sawyer MH, Kennedy M, Williams C, Rhee KE. A brief provider training video improves comfort with recommending the human papillomavirus vaccine. Clinical pediatrics. 2019;58(1):17–23.
13. Garrison A, Fressard L, Mitilian E, Gosselin V, Berthiaume P, Casanova L, et al. Motivational interview training improves self-efficacy of GP interns in vaccination consultations: a study using the Pro-VC-Be to measure vaccine confidence determinants. Human Vaccines & Immunotherapeutics. 2023;19(1):2163809.
14. Kahn JA, Cooper HP, Vadaparampil ST, Pence BC, Weinberg AD, LoCoco SJ, et al. Human papillomavirus vaccine recommendations and agreement with mandated human papillomavirus vaccination for 11-to-12-year-old girls: a statewide survey of Texas physicians. Cancer epidemiology, biomarkers & prevention. 2009;18(8):2325–32.
15. Holder NA, Katzenellenbogen R, Middleman AB. Human papillomavirus vaccines: factors that affect vaccine knowledge and delivery. Journal of adolescent health. 2013;53(3):423–5.
16. Daley MF, Crane LA, Markowitz LE, Black SR, Beaty BL, Barrow J, et al. Human papillomavirus vaccination practices: a survey of US physicians 18 months after licensure. Pediatrics. 2010;126(3):425–33.
17. Statistisches Bundesamt (Destatis). Bevölkerung und Erwerbstätigkeit. Haushalte und Familien – Ergebnisse des Mikrozensus 2021. Erstergebnisse Wiesbaden; 2022.
